# Micro-CT and histological assessment of renal arterial embolization with Glubran®2 cyanoacrylate: a medium-term follow-up study in a rabbit model

**DOI:** 10.1186/s42155-025-00549-8

**Published:** 2025-04-22

**Authors:** Romaric Loffroy, Kévin Guillen, Olivier Chevallier, Mohamed Fouad, Emilie Couloumy, Anne Dencausse, Philippe Robert, Sarah Catoen, Anne-Virginie Salsac, Serge Ludwig Aho-Glele, Pierre-Olivier Comby

**Affiliations:** 1 Department of Vascular and Interventional Radiology, Image-Guided Therapy Center, François-Mitterrand University Hospital, Dijon, 21079 France; 2 ICMUB Laboratory, UMR CNRS 6302, Université de Bourgogne, Dijon, 210000 France; 3 R&D, Guerbet Research, Roissy Charles-de-Gaulle, 95943 France; 4 Biomechanics and Bioengineering Laboratory, UMR CNRS 7338, Université de Technologie de Compiègne, Compiègne, 60203 France; 5 Department of Epidemiology, Statistics and Clinical Research, François-Mitterrand University Hospital, Dijon, 21079 France; 6 Department of Neuroradiology and Emergency Radiology, Image-Guided Therapy Center, François-Mitterrand University Hospital, Dijon, 21079 France

**Keywords:** Transcatheter arterial embolization, N-butyl cyanoacrylate glue, Animal model, Medium-term outcomes

## Abstract

**Background:**

Cyanoacrylate glues are widely used in interventional radiology as effective embolic agents due to their rapid polymerization and ability to achieve vessel occlusion. Nonetheless, concern remains regarding cast stability and potential recanalization over time. This study used multiple modalities to evaluate the medium-term outcomes of Glubran®2 glue (methacryloxysulfolane and N butyl cyanoacrylate) embolisation in a rabbit renal-artery model.

**Methods:**

The left renal arteries of six rabbits were embolized with 12.5% or 25% Glubran®2. In-vivo micro-CT scans were performed immediately after embolisation (M0) and ex-vivo scans and a histological assessment were done at one month (M1). Magnetic resonance imaging (MRI) was done at M1 to assess arterial occlusion and parenchymal changes. Quantitative and semi-quantitative parameters reflecting glue distribution, cast integrity, and tissue response were analysed. Statistical comparisons used non-parametric tests.

**Results:**

All six embolisations were completed without complications. Micro-CT at M1 revealed significant cast resorption and fragmentation with both concentrations, but with no evidence of arterial recanalization. MRI and histology confirmed the persistent vascular occlusion with chronic ischemic changes in the renal parenchyma. Compensatory neovascularization from the renal capsule was observed, with no significant differences in histological inflammation between the two concentrations. Glue casts remained within the arterial lumens and were often surrounded by granulomatous inflammation.

**Conclusions:**

Glubran®2 was effective for renal artery embolisation, even at a low concentration of 12.5%: despite partial cast resorption, the arteries remained occluded. Micro-CT proved to be a powerful tool for assessing changes in glue casts. Longer-term studies are warranted to further assess vascular remodelling and occlusion durability.

## Background

Cyanoacrylate glues are widely used for transcatheter arterial embolisation because they polymerize promptly upon contact with body fluids, thereby providing prompt vessel occlusion [1]. This rapid effect is crucial in several indications such as arteriovenous malformations (AVMs) and arterial bleeding [2, 3]. The histological effects of cyanoacrylate glues have been extensively documented [4, 5]. Acute vascular inflammation is followed by a foreign-body granulomatous reaction with macrophage infiltration and the formation of multinucleated giant cells [6, 7].

Although cyanoacrylate glues have been proven effective in achieving vessel occlusion, evidence of partial cast resorption over time after AVM embolisation has raised concern about the durability of the occlusive effect, independently of the number of supply arteries [8]. Whether this partial cast resorption affects the degree of occlusion or the risk of recanalization remains unclear, even if resorption does not mean recanalization in our experience.

Microcomputed tomography (micro-CT) provides accurate three-dimensional images of glue casts within blood vessels [9]. Cast distribution and changes over time can be assessed.

The primary objective of this animal-model study was to perform micro-CT assessments of changes in glue casts over the first post-embolisation month and to compare micro-CT findings with histological findings at one month. The secondary objective was to look for histological evidence of persistent arterial occlusion at one month.

## Methods

### Animal model and embolisation procedure

The study protocol was approved by the local animal experimentation ethics committee (CEEA n°106, #25,592) on 23rd June 2020. We studied six, 3-month-old, female rabbits obtained from Charles River (Écully, France). For the procedure, the animals were sedated with intramuscular buprenorphine (Buprecare® 0.3 mg/mL, 0.17 mL/kg; Animalcare, York, UK) then anaesthetised with 2% isoflurane (IsoFlow®, Zoetis, Parsippany-Troy Hills, NJ, USA).

Two interventional radiologists (POC and KG) performed the procedures using a monoplane angiographic system (Veradius, Philips, Amsterdam, The Netherlands). A 4-Fr introducer was inserted surgically into the femoral artery, meaning by direct puncture after cutdown given the small vessel size. A baseline angiogram of the abdominal aorta was obtained using a 4-Fr vertebral catheter and manual injection of the water-soluble contrast agent iobitridol (Xenetix 350, Guerbet, Villepinte, France) via a 10-mL syringe, at a flow rate of about 4 mL/s. A 2.3-Fr microcatheter (Phenom™ 21, Medtronic, Dublin, Ireland) was then inserted co-axially into the 4-Fr catheter in the left renal artery. Special care was taken to avoid selecting ventral or dorsal branches and to ensure that the glue was injected under free-flow conditions.

To prevent premature glue polymerisation, the microcatheter was first flushed with 5% glucose in a volume of at least 0.43 mL, i.e., at least the volume of the lumen. The glue-LUF mixture was injected under fluoroscopic guidance using a 5-mL syringe (Plastipak™, Becton Dickinson Plastic, Franklin Lakes, NJ, USA) and an automated syringe pump (Harvard Apparatus PHD 2000™, Holliston, MA, USA) at a standardised flow rate of 0.03 mL/s, as in previous studies [9, 10].

Embolisation was deemed complete when reflux along the microcatheter was detected or the glue ceased to advance within the vascular tree. Either of these signs led to immediate withdrawal of the microcatheter to avoid adhesion to the vessel wall. The injected volume was computed by subtracting the volume of the microcatheter lumen (0.43 mL) from the total injected volume. The duration of the injection was recorded for each procedure.

The 4-Fr introducer was removed and the puncture site was closed with sutures.

### Embolic agent

All procedures were done using Glubran®2 glue (N-butyl-2-cyanoacrylate [NBCA] plus methacryloxysulfolane, GEM SRL, Viareggio, Italy) mixed manually with Lipiodol® Ultrafluid (LUF, Guerbet) at room temperature. We studied two glue concentrations, 12.5% (glue/LUF ratio, 1:7) and 25% (1:3). The mixtures at each of the two concentrations were injected under free-flow conditions into the left renal arteries of three animals.

### Micro-CT evaluation

Each rabbit underwent an in-vivo micro-CT scan immediately after embolisation (M0). One month later (M1), the animals were euthanised and the left kidney of each was removed and imaged ex-vivo by micro-CT.

#### In-vivo image acquisition

Immediately after embolisation, the rabbit was transported to the micro-CT room for in-vivo imaging while continuously anaesthetised using a 2% isoflurane mask (Zoetis).

**Fig. 1:**
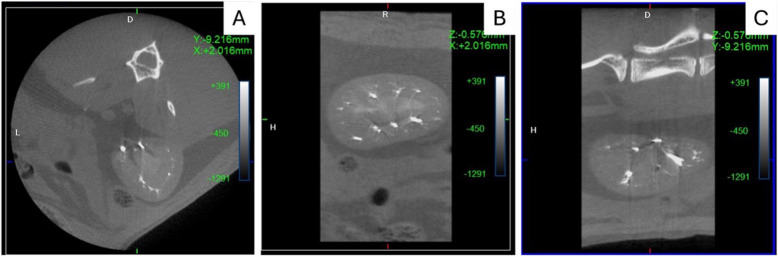
In-vivo micro-CT images obtained immediately after embolization (M0). Axial (**A**), coronal (**B**), and sagittal (**C**) reconstructions

We used a CosmoScan GX (Rigaku Analytical Devices, Tokyo, Japan) equipped with a dedicated small-animal camera. The settings for each acquisition were as follows: 90 kV, 88 μA, field-of view 72 mm, pixel size 144 μm, and acquisition duration 4 min. Image reconstruction was with both soft and hard filters. ViVoquant™ software (Invicro, Needham Heights, MA, USA) was used for post-processing. Figure 1 shows the in-vivo micro-CT images of a rabbit embolized with 12.5% Glubran®2.

#### Ex-vivo image acquisition

The micro-CT component of the NanoSPECT/CT Plus small-animal camera (Bioscan, Washington DC, USA) was used for imaging. Acquisition parameters were as follows: 55 kV; exposure time, 1000 ms for each of the 240 projections; axial range, 40–50 mm; and step size, 1. The scan acquisition time was 6 to 9 min. Bioscan image processing software was used for image reconstruction, with application of a cone-beam filtered-backprojection algorithm to generate image slices with a voxel size of 36 × 36 × 73 μm^3^. Each reconstructed slice was post-processed using ViVoquant™ software (Invicro).

#### Objective evaluation: quantitative parameters

To assess depth of glue penetration, we measured the cast-to-capsule distance (mm) at the upper pole, mid-region, and lower pole of each kidney. Segmentation started at the first bifurcation of the renal artery. This technique minimises variability in cast volume measurement due to differences in the length of the extra-renal artery included in the sample [6]. Cast segmentation was based on the attenuation level with three categories (1000–1300 HU, > 1300–1600 HU, and > 1600 HU) chosen to account for the high attenuation of LUF, which can create artifacts.

To reduce variability due to differences in animal characteristics such as weight and age, we computed the ratio of cast volume over renal parenchyma volume. A cast attenuation ≥ 1300 HU was selected for comparing ratios, based on our previous experience [9].

#### Subjective evaluation: semi-quantitative parameters

##### Image quality

To assess image quality across the different micro-CTs, we used a 5-point Likert scale to measure noise (1, unacceptable; 2, above-average; 3, average; 4, below-average; and 5, minimal). Disagreements were resolved by consensus.

##### Embolisation assessment

We used a semi-quantitative scale to assess depth of glue penetration: 1, main renal artery and its first branches; 2, interlobar artery; 3 corticomedullary junction; 4, deep cortical interlobular arteries; and 5, superficial cortical interlobular arteries. Cast fragmentation was scored 0 (none), 1 (slight), or 2 (marked). Cast heterogeneity, defined as the presence of decreased attenuation within the glue cast, was evaluated in the first three of the five above-described penetration zones using a 0–4 scale (0, homogeneous; and 1, 2, 3, and 4, hypoattenuation in < 25%, 25–50%, 50–75%, and ≥ 75% of the cast, respectively). Cast heterogeneity proved difficult to assess in the small distal arteries and we consequently used only the scores for the first two glue-penetration zones in the statistical analysis. Finally, we assessed overall cast resorption on the micro-CT at one month, as follows: 0, none; 1, moderate; and 2, marked.

### Magnetic resonance imaging

MRI scans were performed immediately after embolisation (M0) and one month later, just before the animal was euthanised, to assess the persistence of arterial occlusion over time and the changes in the parenchyma. A Biospec 4.7 T preclinical MRI (Bruker, Billerica, MA, USA) machine adapted for small animals was used with 2D coronal T1 ultra-fast dynamic sequences acquired at the early arterial phase to visualize progression of the contrast bolus along the vascular tree. The images were reconstructed using 3DSlicer software (V5 slicer.org).

### Histopathological evaluation

One month after the embolisation, the animal was euthanised under general anaesthesia with an intravenous bolus of Euthasol (0.34 mL/kg, Med-Vet International, Mettawa, IL, USA). Each renal artery/kidney unit was harvested and rapidly fixed in 10% neutral buffered formalin solution. Fixed samples were then sectioned and embedded in paraffin. Longitudinal sections of each kidney at two different levels were prepared and stained with haematoxylin–eosin. The 12 resulting histological slides were evaluated by a pathologist blinded to the characteristics of the procedure.

For each slide, the pathologist assessed arterial lumen dilation (0, none; 1, mild; 2, moderate; 3, severe). Intimal arteritis was defined as the presence of inflammatory cells, primarily lymphocytes and monocytes, in the subendothelial compartment of one or more arteries and was quantified using the Banff 97 scale (0, no arteritis; 1, mild intimal arteritis; 2, marked intimal arteritis with loss of ≥ 25% of the luminal area; and 3, transmural arteritis and/or fibrinoid arterial changes [11, 12]. Intimal necrosis was evaluated as follows: 0, none; 1, focal intimal necrosis with smooth muscle vacuolisation; and 2, multifocal necrosis with expanded and flattened intima. Arterial occlusion by glue was assessed as 0, no occlusion; 1, incomplete occlusion; 2, nearly complete occlusion; and 3, complete occlusion. The percentage of necrotised parenchyma was calculated. Glue penetration was evaluated based on the five above-described penetration zones. For each parameter and each kidney, the highest value was collected. Whether revascularization had occurred from the capsule was documented, and the thickness of the renal parenchyma occupied by newly formed blood capillaries was measured (μm) from the capsule surface.

**Fig. 2:**
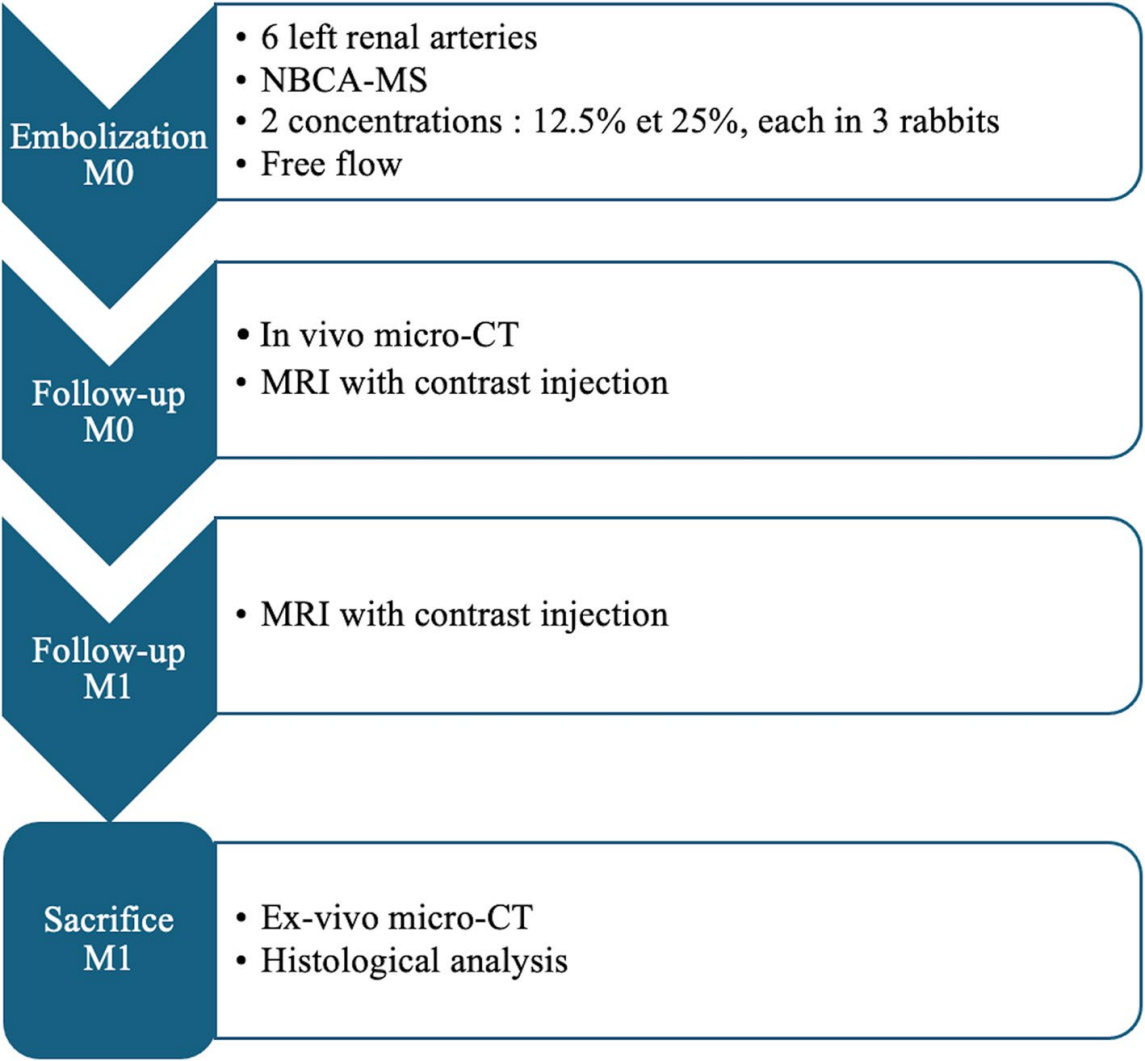
Diagram of the experimental protocol

Figure 2 recapitulates the assessment protocol.

### Statistical analysis

The Shapiro–Wilk test showed a skewed distribution for some variables. We therefore described all variables as median [interquartile range] (range) [13]. Semi-quantitative variables (e.g., arterial lumen dilation, intimal arteritis, intimal necrosis, and glue penetration) were handled as ordinal variables. To compare the micro-CT parameters between M0 and M1 in each rabbit, given the small sample size, we chose the non-parametric Wilcoxon test. The Mann–Whitney test was applied to compare histological data at M0 and M1. Values of *p* ≤ 0.05 were considered statistically significant. Statistical analyses were performed using DATAtab: DATAtab Team (2024) (Graz, Austria, https://datatab.net).

## Results

### Embolisation procedures

The renal arteries of six rabbits were embolized using either of the two glue concentrations. No instances of microcatheter adhesion to the vessel walls or other intra-procedural complications occurred. The median weight of the rabbits was 3.05 [2.68–3.67] kg. There were no significant differences between the two glue-concentration groups regarding weight, glue volume injected, or injection duration. Table 1 lists the characteristics of the rabbits and procedures.

**Table 1:** Characteristics of the six rabbits and embolisation procedures

	**NBCA-MS 12.5%**	**NBCA-MS 25%**	**Total**	***p* value**
Embolised arteries, n (%)	3 (50)	3 (50)	6 (100)	> 0.99
Parenchymal histological sections, n (%)	6 (50)	6 (50)	6 (100)	> 0.99
Rabbit weight (kg), median [IQR] (range)	3.1 [2.89–3.39] [2.68–3.67]	3 [2.96–3.1] [2.91–3.19]	3.05 [2.93–3.17] [2.68 − 3.67]	0.17
Injected glue-mixture volume (mL), median [IQR] (range)	0.23 [0.22–0.40] (0.22–0.58)	0.25 [0.18–0.25] (0.11–0.26)	0.24 [0.22–0.25] (0.11 − 0.58)	0.42

*NBCA-MS* N-butyl cyanoacrylate methacryloxysulfolane

### Imaging results

#### Comparison of Micro-CT results at M0 and M1

##### Quantitative parameters

The cast-to-kidney ratio ranged from 0.04% to 30.49%, and the cast-to-capsule distance from 0.08 mm to 4.38 mm. The cast-to-kidney ratios decreased significantly from M0 to M1. The cast-to-capsule distance showed a non-significant decrease between M0 and M1. Results are summarized in Table 2.

**Table 2:** Micro-CT findings immediately after embolization (M0) and one month later (M1)

	**Total**			**NBCA-MS 12.5%**		**NBCA-MS 25%**	
**Findings**	**M0**	**M1**	***p* values**^**a**^	**M0**	**M1**	**M0**	**M1**
**Semi-quantitative parameters, n (%)**							
** Image quality, Likert scale rating**							
2	1 (17)	0	**0.014**	1	0	0	0
3	5 (83)	0		2	0	3	0
4	0 (0)	1 (17)		0	1	0	0
5	0 (0)	5 (83)		0	2	0	3
** Distality of glue penetration, n (%)**							
Interlobular arteries of the deep cortex	5 (83)	1 (17)	**0.046**	2	1	3	0
Interlobular arteries of the superficial cortex	1 (17)	5 (83)		1	2	0	3
** Cast fragmentation, n (%)**							
Slight	5 (83)	0 (0)	**0.025**	3	0	2	0
Marked	1 (17)	6 (100)		0	3	1	3
**Cast heterogeneity score, n (%)**							
** Renal artery and first branches**							
> 50% and ≤ 75%	NA	3 (50)	NA	NA	1	NA	2
75% to 100%	NA	3 (50)		NA	2	NA	1
** Interlobar artery**							
> 25% and ≤ 50%	NA	1 (17)	NA	NA	1	NA	0
> 50% and ≤ 75%	NA	2 (33)		NA	0	NA	2
75% to 100%	NA	3 (50)		NA	2	NA	1
** Overall resorption, n (%)**							
Moderate	NA	2 (33)	NA	NA	1	NA	1
Marked	NA	4 (66)		NA	2	NA	2
** Quantitative parameters, median [IQR] (range)**							
** Cast-to-capsule distance (mm)**	2.06 [1.6–2.74] (1.49 − 4.38)	0.75 [0.29–1.31] (0.08 − 2.47)	0.116	1.56 [1.53–1.64] (1.49 − 41.71)	0.91 [0.5–1.69] (0.08 − 2.47)	2.85 [2.64–3.62] (2.42 − 4.38)	0.25 [0.39–1.01] (0.59 − 0.18)
** Indexed cast ratio (%)**	10.6 [8.53–12.66] (1.16 − 30.49)	0.08 [0.05–0.53] (0.04 − 1.29)	**0.028**	12.79 [10.59–21.64] (8.38 − 30.49)	0.06 [0.06–0.08] (0.05 − 0.1)	8.96 [5.06–10.6] (1.16 − 12.24)	0.67 [0.36–0.098] (0.04 − 1.29)

^a^Wilcoxon test

*NBCA-MS* N-butyl cyanoacrylate methacryloxysulfolane, *NA* not available

##### Semi-quantitative parameters

Image-quality Likert-scale scores for the micro-CTs were 2 or 3 at M0 and 4 or 5 at M1 (*p* = 0.014). Given the higher noise levels at M0, probably ascribable to respiratory movements, cast heterogeneity was not evaluated at this time point. At M1, glue was seen in zone 5 (superficial interlobular arteries) in five kidneys and in zone 4 (deep interlobular arteries) in one kidney. Penetration was significantly more distal at M1 than at M0.

Cast fragmentation was observed in all six kidneys at both M0 and M1 and was significantly more marked at M1 than at M0 (*p* = 0.025). Results are summarized in Table 2.

**Fig. 3:**
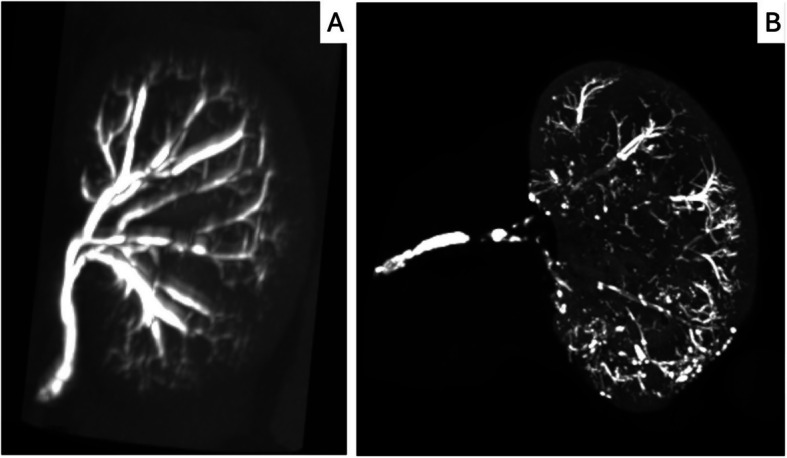
Maximum intensity projection reconstructions of micro-CT scans of the same rabbit kidney embolised with 12.5% Glubran.®2. **A** immediately after embolization (M0), and (**B**) one month later (M1)

Figure 3 shows micro-CT images at M0 and M1 of a kidney embolized with 12.5% NBCA-MS.

#### Magnetic resonance imaging

**Fig. 4:**
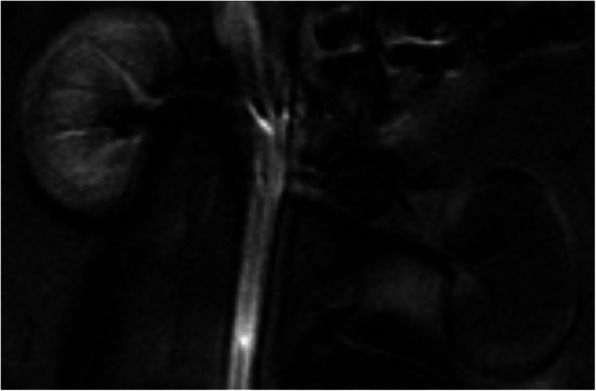
MRI done immediately after embolization (M0) of the left renal artery with 25% Glubran®2. Coronal T1 view after contrast injection at the arterial phase showing occlusion of the left renal artery with no enhancement of the left renal parenchyma

The MRIs done immediately after embolisation (M0) showed evidence of sudden parenchymal infarction indicating arterial occlusion: size was normal and the parenchyma showed no enhancement at the arterial or venous phase. Figure 4 shows an example.

**Fig. 5:**
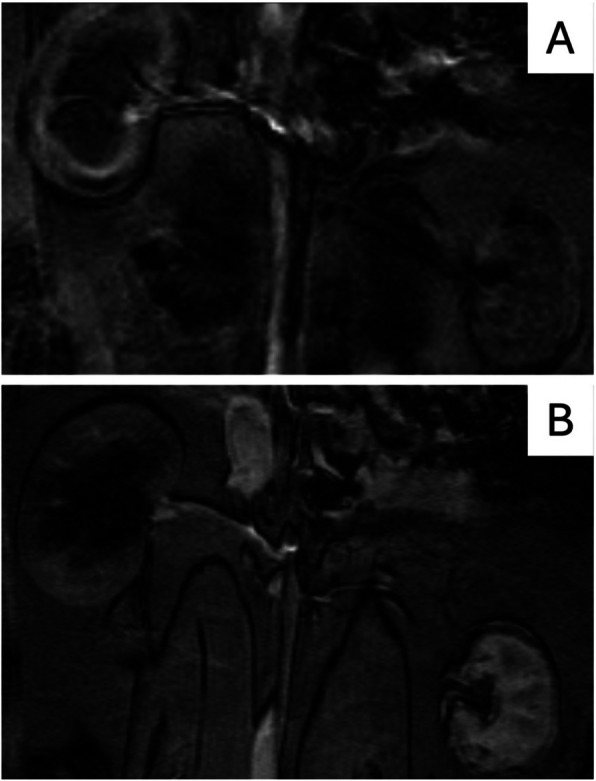
MRI done one month (M1) after embolization of the left renal artery with 25% Glubran®2. **A** coronal T1 view after contrast injection at the early arterial phase: persistent occlusion of the left renal artery with no parenchymal enhancement. **B** coronal T1 view at the late venous phase: parenchymal enhancement due to subcapsular neovascularisation

The findings from the in-vivo MRIs at M1 were markedly different from those at M0, with typical signs of chronic renal ischemia and parenchymal shrinkage. The contrast-enhanced sequences visualised delayed parenchymal enhancement related to neovascularisation from the capsule. Figure 5 shows an example.

### Histological findings

#### Glue

In all 12 samples, glue remnants were visible as amorphous plugs of eosinophilic material confined to the vascular lumen. No glue was detected outside the lumens. Granular aggregates of greyish to bluish material were seen occasionally, probably due to loss of small amounts of glue, consistent with the lack of tissue anchorage and the frequent presence of optically empty areas within the dilated vascular lumens.

#### Alterations in arterial lumen and wall

**Fig. 6:**
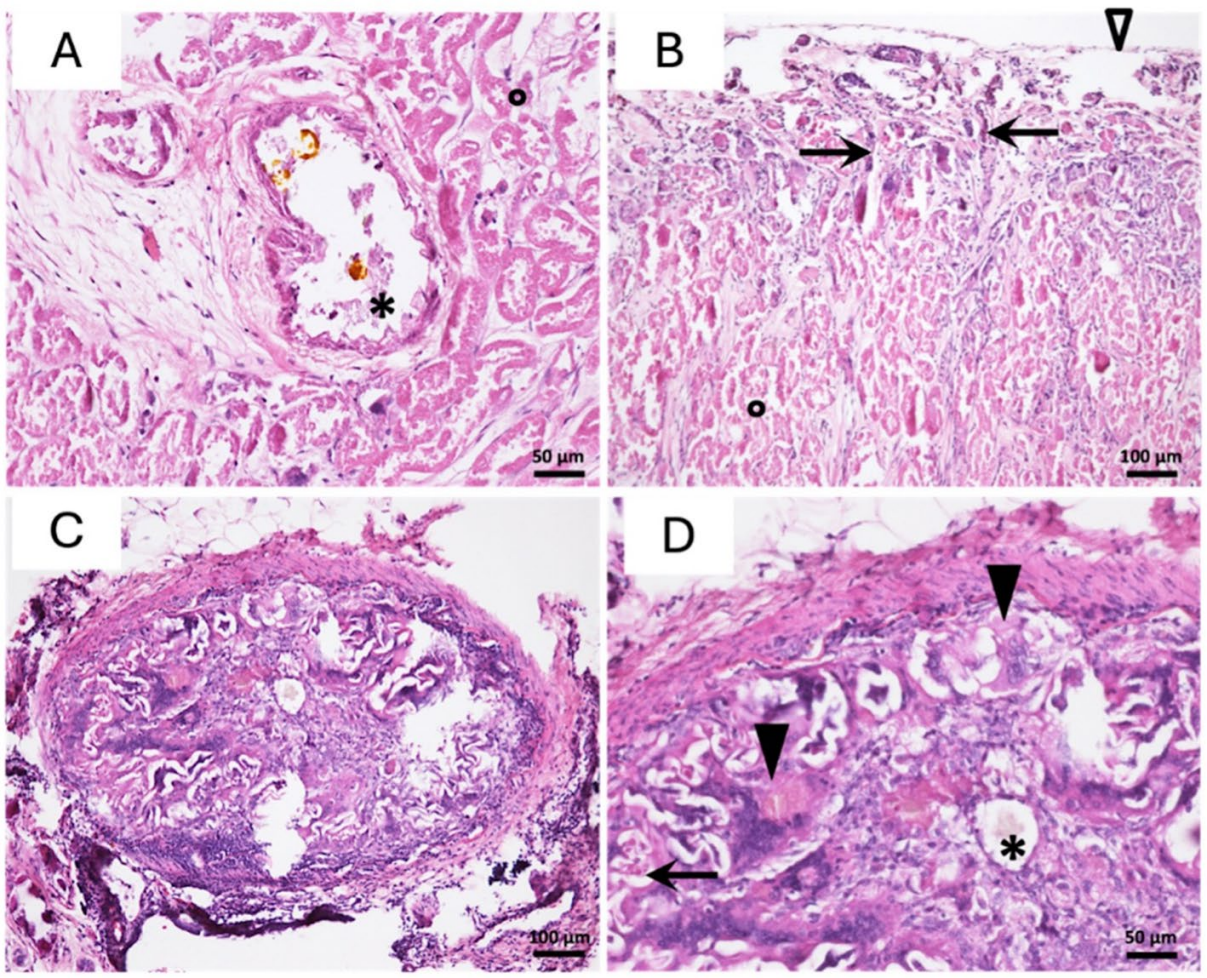
Main histological findings in the embolised kidneys (haematoxylin–eosin stain). The arteries containing glue (*) were obstructed and dilated, and their walls were necrotic and flattened. Coagulative necrosis was visible in most of the renal parenchyma, with preservation of the normal architecture and ghost-like necrotic cells (°) and only a few preserved areas (top left panel). Small neocapillaries ( →) were visible focally beneath the renal capsule (∆), indicating revascularisation (top right panel). Glue plugs completely obstructing some of the large vessels were surrounded by inflammatory granulomas (bottom left panel) containing activated macrophages and multinucleated giant cells (▲). A few neocapillaries with large lumens ( →) were present focally, again indicating reperfusion (bottom right panel)

Lumen dilation was observed in all 12 samples and was severe in five. All 12 samples exhibited transmural arteritis, intimal necrosis, and complete or nearly complete occlusion of the arterial lumen. Results are summarized in Table 3. Figure 6 shows examples of the main histological findings.

**Table 3:** Histological findings in the six embolised kidneys (two slides per kidney)

Findings	NBCA-MS 12.5%	NBCA-MS 25%	Total	*p* value^a^
**Arterial lumen dilation, n (%)**				
Mild	4 (33)	3 (25)	7 (58)	0.39
Moderate to severe	2 (17)	3 (25)	5 (42)	
**Intimal arteritis, n (%)**				
Transmural	6 (50)	6 (50)	12 (100)	> 0.99
**Intimal necrosis, n (%)**				
Multifocal	6 (50)	6 (50)	12 (100)	> 0.99
**Arterial occlusion, n (%)**				
Nearly complete	3 (25)	2 (17)	5 (42)	0.70
Complete	3 (25)	4 (33)	7 (58)	
**Distality of glue penetration**				
Interlobular arteries of the deep cortex: zone 4	2 (17)	4 (23)	6 (50)	0.31
Interlobular arteries of the superficial cortex: zone 5	4 (33)	2 (17)	6 (50)	
**Parenchymal inflammation, n (%)**				
Mild	5 (42)	2 (17)	7 (58)	0.13
Marked	1 (8)	4 (33)	5 (42)	
**Necrotic parenchyma (% of total parenchyma, median [IQR] (range)**	90 [90–90] (80–90)	95 [90–100] (90–100)	90 [90–92.5] (80–100)	0.093
**Revascularization from the capsule (μm), median [IQR] (range)**	985 [792–1072) (350 − 1080)	850 [700–1075] (350 − 1480)	960 [700–1080] (350 − 1480)	0.94

^a^Mann-Whitney test

*NBCA-MS* N-butyl cyanoacrylate methacryloxysulfolane

#### Glue penetration

In all six kidneys, glue was observed in the interlobular arteries of the deep or superficial cortex. The glue penetrated to the mid or distal parts of the interlobular arteries (score 5) in six samples. In the remaining six samples, the score was 4, with glue present in the arcuate arteries or the proximal part of the interlobular arteries.

#### Inflammation and host tissue reaction

For most of the medium and small arteries, no reactive changes were seen in the tissue surrounding the glue casts. In contrast, the glue remnants within large, obstructed arteries (i.e., chiefly the lobar and interlobular arteries) were surrounded by a pronounced granulomatous reaction, with lymphocytes and numerous macrophages, some of which formed multinucleated giant cells, creating a foreign body-type granuloma.

#### Renal parenchymal changes

Arterial occlusion by glue was associated with extensive necrosis of the renal parenchyma. The necrosis was sometimes surrounded by small areas of mononuclear inflammatory-cell infiltration and interstitial fibrosis. In the medulla, some of the parietal tubular cells appeared hyperbasophilic with a large euchromatic nucleus, indicating active tubular regeneration. The renal capsule was thickened in some areas, and revascularization from the capsule towards the cortex was observed. The thickness of the renal parenchyma occupied by blood capillaries, measured from the capsule, varied from 350 to 1480 μm.

None of the histological parameters showed significant differences between the two glue concentrations (12.5% vs. 25%). Detailed results are provided in Table 3.

## Discussion

Micro-CT imaging of Glubran®2 casts within the renal vascular system of rabbits detected notable changes over the first month following embolisation. Various degrees of partial resorption manifesting chiefly as increased fragmentation compared to baseline were seen in all kidneys. Partial resorption was similar with the 12.5% and 25% Glubran®2 concentrations. Importantly, in no instance did this partial resorption result in arterial recanalization: the MRI and histological findings documented the persistence of complete vessel occlusion.

Micro-CT scanning provided accurate information on the consequences of Glubran®2 embolisation. To our knowledge, our study provides the first data on glue-cast changes over time. As expected, we found signs of renal infarction, with MRI evidence of parenchymal shrinkage predominating in the cortex [14]. The significant decrease in the cast-to-parenchyma volume ratio between M0 and M1 is consistent with a faster pace of cast resorption than of parenchymal atrophy. Alternatively, residual contrast material not yet excreted via the urinary tract may have resulted in cast-volume overestimation by the in-vivo segmentation calculations. In future studies, micro-CT may provide valuable data on vessel remodelling after glue embolisation.

A major strength of our study is the assessment of a low Glubran®2 concentration, of 12.5%. Low concentrations are used in emerging clinical applications such as prostatic artery embolisation. In a retrospective cohort study of 103 patients with benign prostatic hyperplasia, a glue/LUF ratio of 1:8 was used, corresponding to an absolute glue concentration of 11.1%, to facilitate greater distality of penetration [15]. Good 6-month outcomes were documented. In the present study, even the low concentration of 12.5% ensured persistent arterial occlusion without cast recanalisation at M1. The histological evidence of inflammation was not significantly different between the two concentrations, in keeping with a previous study [16].

Neocapillaries developed from the renal capsule during the 1-month follow-up, as documented histologically and by the late-phase parenchymal enhancement on the MRI at M1. The degree of neovascularisation was not significantly different between the two glue concentrations.

The partial resorption of the glue over the 1-month follow-up is consistent with data on the long-term degradation of NBCA after embolisation, which occurs via several mechanisms. First, hydrolysis of the cyanoacrylate ester group partially fragments the polymer. The by-products of this hydrolysis, such as formaldehyde, contribute to the local inflammation and gradual glue resorption [17]. Second, the prolonged presence of glue within tissues causes an immune response with a foreign-body-type inflammatory process [18]. A granuloma consisting of macrophages and multinucleated giant cells develops around the glue and releases lysosomal enzymes that contribute to cast resorption. Finally, fibrotic scar tissue builds up gradually, encapsulating the glue and contributing to its resorption [19]. The combination of these three mechanisms results in gradual disappearance of the glue cast.

One major limitation of our study is the small sample size, with only three rabbits in each glue-concentration group, which limits the statistical power of our study and the general applicability of our findings. Furthermore, we only used the kidney as an embolic location and did not consider other sites. However, the 3Rs principles for maximising animal welfare include the use of the smallest possible number of animals, and this location allowed easy surgical removal for ex-vivo imaging by micro-CT. Second, the predominantly terminal nature of the renal vasculature may affect the durability of arterial occlusion. In addition, the kidney is more sensitive to ischaemia but this sensitivity is more reproducible compared to other organs. Third, we used different micro-CT machines for the in-vivo scanning at M0 and the ex-vivo scanning at M1. Fourth, the resolution of in-vivo scans was slightly lower than that of the ex-vivo scans. As a result, cast heterogeneity was evaluable on the ex-vivo but not the in-vivo images. The lower resolution in vivo is probably ascribable to the respiratory movements. Optimisation of micro-CT acquisition protocols might produce sufficient resolution to assess cast heterogeneity in vivo.

## Conclusions

By combining micro-CT, MRI, and histology, we obtained detailed information about changes in Glubran®2 casts over the first month following embolisation. We documented substantial cast resorption over time, with fragmentation and dissolution in the renal parenchyma. Importantly, the histological assessments showed that cast resorption did not affect arterial occlusion, even with the low Glubran®2 concentration of 12.5%. Longer-term studies are needed to further characterise the durability of arterial occlusion by Glubran®2.

## Data Availability

The datasets used and/or analysed during the current study are available from the corresponding author on reasonable request.

## Abbreviations

AVM: Arterio-venous malformation
LUF: Lipiodol Ultra Fluid
M: Month
NBCA: N-butyl cyanoacrylate
